# The breast cancer genome - a key for better oncology

**DOI:** 10.1186/1471-2407-11-501

**Published:** 2011-11-30

**Authors:** Hans Kristian Moen Vollan, Carlos Caldas

**Affiliations:** 1 Department of Genetics, Institute for Cancer Research and Department of Breast and Endocrine Surgery, Division of Surgery and Cancer, Oslo University Hospital Radiumhospitalet, 0310 Oslo, Norway; 2 Institute of Clinical Medicine, Faculty of Medicine, University of Oslo, 0318 Oslo, Norway; 3 Breast Cancer Functional Genomics, Cancer Research UK Cambridge Research Institute, Cambridge, UK; 4 Department of Oncology, University of Cambridge, Li Ka-Shing Centre, Robinson Way, Cambridge CB2 0RE, UK; 5 Cambridge Breast Unit, Addenbrooke's Hospital and Cambridge National Institute for Health Research Biomedical Research Centre, Cambridge University Hospitals NHS Foundation Trust, Hills Road, Cambridge CB2 0QQ, UK

## Abstract

Molecular classification has added important knowledge to breast cancer biology, but has yet to be implemented as a clinical standard. Full sequencing of breast cancer genomes could potentially refine classification and give a more complete picture of the mutational profile of cancer and thus aid therapy decisions. Future treatment guidelines must be based on the knowledge derived from histopathological sub-classification of tumors, but with added information from genomic signatures when properly clinically validated. The objective of this article is to give some background on molecular classification, the potential of next generation sequencing, and to outline how this information could be implemented in the clinic.

## Molecular classification of breast cancer

The diversity of breast cancer has been acknowledged for decades, but recent technological advances in molecular biology have given detailed knowledge on how extensive this heterogeneity really is. Traditional classification based on morphology has given limited clinical value; mostly because the majority of breast carcinomas are classified as invasive ductal carcinomas, which show a highly variable response to therapy and outcome [1]. The first molecular sub-classification with a major impact on breast cancer research was proposed by Perou and colleagues where the tumors were subdivided according to their pattern of gene expression [2,3]. Five groups were identified and named Luminal A, Luminal B, Basal-like, Normal-like and the HER-2-enriched subgroups. These intrinsic subgroups have been shown to be different in terms of biology, survival and recurrence rate [3,4]. The molecular subgroups have been extended to also include a sixth subgroup which has been named the claudin- low group, based on its low expression level of tight junction genes (the claudin genes) [5]. Different methods for the assignment of individual tumors to its molecular subgroup is proposed; each based on the expression levels of different sets of genes [4,6,7]. The agreement between methods on how to classify individual tumors are not optimal and how to establish more robust single sample predictors is actively debated [8-11].

Aneuploidy is the presence of an abnormal number of parts of or whole chromosomes and is one feature that clearly separates cancer cells from normal cells. This was proposed as being important in cancer nearly a century ago by Theodor Boveri [12]. With array-based comparative genomic hybridization (aCGH) a genome wide profile of the copy number alterations in the tumor can be obtained. These patterns are related to the molecular subtypes with distinct differences in the number of alterations between the subtypes [13-16]. These copy number alterations (CNAs) alter the dosage of genes and highly influence the level of expression [17,18]. This frequently affects the activity in oncogenes and tumor suppressor genes and in this way CNAs are important for the carcinogenic process. CNAs in tumors are a result of deregulated cell cycle control and of DNA maintenance and repair [19]. Different patterns of copy number alterations have been identified with distinct differences; simplex profiles are characterized by few alterations and complex genomic profiles have extensive changes [20]. Complex genomic rearrangements are areas with high-level amplifications and have prognostic value in breast cancer even when they do not harbor known oncogenes, suggesting that the phenotype of defect DNA-repair may be associated with more aggressive disease [20,21].

Alterations in the expression pattern are caused by changes at the genomic level and a robust classification of breast cancer for clinical use should probably take these more into account. Changes at the genomic level include point mutations, changes in copy number and epigenetic events. These are characteristics that enable and drive carcinogenesis together with tumor-promoted inflammation [22].

## The era of sequencing of cancer genomes

**Figure 1:**
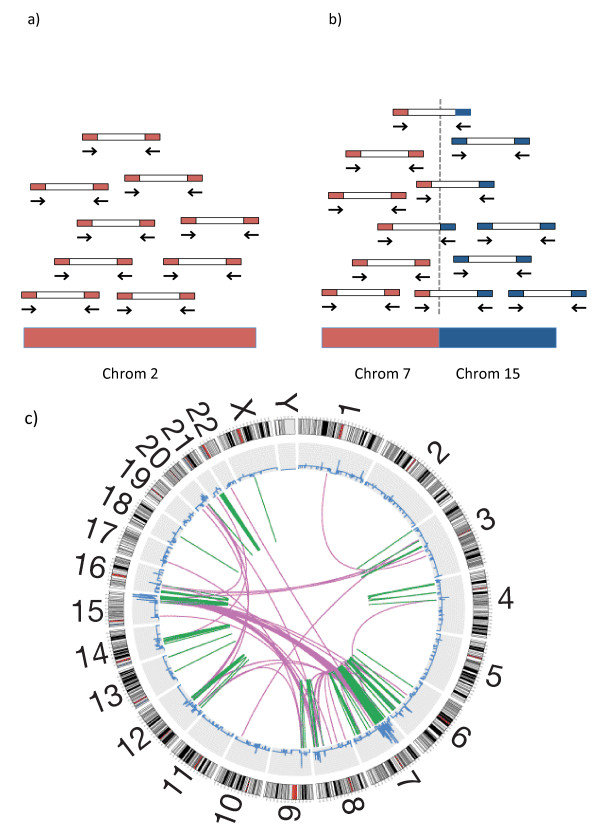
**The basis of translocation mapping from paired-end sequencing**. **(a) **Paired end sequencing is based on sequencing a short sequence of nucleotides of each end of fragmented and amplified genomic DNA. Reads without the desired length are filtered out. All reads are aligned to a reference genome. The average number of reads per genomic locus is called the coverage of the genome of the sequenced sample. A high coverage (20× to 40×) is needed for detection of point mutations while a much lower coverage is required for other analysis such as copy number and mapping of translocations. The number of reads that map to a locus can be regarded as a function of the number of copies of that locus. As reads can be binned across windows the coverage does not need to be high for such analyses. **(b) **When a part of a chromosome is fused to a part of another chromosome the read from this region will have a sequence in one end that maps to one chromosome and the other end maps to another. When this pattern is consistent over several reads the translocations can be precisely mapped. Intrachromosomal rearrangements are mapped the same way. **(c) **A circos plot of a breast cancer genome. The chromosomes are arranged as a circle from chromosome 1 to the sex chromosomes X and Y. The outer part of the circle shows the chromosomes with cytoband information. The blue line represents the copy number at the given loci. The lines in the middle represent translocations. The inter-chromosomal translocations are in purple and the intra-chromosomal translocations are shown in green. Part (c) is modified from Russnes *et al*. [21].

We are now in the exciting era of full sequencing of cancer genomes. Paired-end sequencing is based on massive parallel sequencing of short stretches of nucleotides at each end of fragmented DNA [23]. The basis of paired-end sequencing technology is shown in Figure 1. Next generation sequencing gives additional information to cancer genomics at many levels, including point mutations, insertions, deletions, copy number and translocations depending on the level of the coverage [23]. The copy number alterations in breast cancer are well characterized by aCGH, but sequencing has given important insight into how alterations are structured given that information on translocations/rearrangements is added [24].

Stephens *et al*. described multiple rearrangement architectures after sequencing 9 breast cancer cell lines and 15 tumors [24]. Intrachromosomal rearrangements were found to be far more frequent than between chromosomes and the most common event was tandem duplications, but with a high degree of variation among tumors. They hypothesized that these extensive alterations are a consequence of a DNA repair defect that leads to a 'mutator phenotype' similar to what causes microsatellite instability in other cancers. Breakpoints tended to fall into areas with microhomology and non- template sequences. Fusion genes are hybrid genes formed from two separate genes (for example, by translocations), which can lead to functional proteins with oncogenic properties. These are important in leukemias and lymphomas, but the role of fusion genes in breast cancer is unclear [25]. Stephens *et al*. found enrichment for alterations within genes and 29 of these were predicted to generate in-frame gene fusions. Transcripts were found for 21 of these, but none of these were recurrent among cancers [24]. Sequencing of the cell line MCF-7 has revealed that breakpoints that are evenly dispersed over the genome tend to be in areas of low copy repeats while the more clustered breakpoints occur close to high-level amplified genes, pointing to different mechanisms for genomic instability [26]. Important point mutations are present already at an early stage, as has been shown in a comparative deep sequencing study of the genomes, and transcriptomes of a primary lobular tumor and its distant metastasis 9.5 years later [27].

The sequencing technology is now capable of sequencing genomes of single cells. As there are heterogeneity among cells of the tumor and infiltration of normal cells and inflammatory cells, picking the right cell to sequence may be challenging. Navin *et al*. sequenced 100 single cells from a polygenic tumor that revealed four distinct groups of genomes; the diploids and the pseudo-diploids (representing normal cells and immune cells), one hypo-diploid and two aneuploid groups [28]. Their analysis suggests that these represent three clonal expansions in the primary tumor as they share many common aberrations. A total of 52 cells from a second tumor and 48 cells from a paired liver metastasis were sequenced and the results indicated that a clonal expansion from a single aneuploid cell had formed the primary tumor and that one of these had metastasized to the liver forming the metastasis.

Deep sequencing of cancer genomes is a costly process and the amount of biological material needed has been a challenge, but technology is moving fast and both cost and tissue demands are continuously decreased. International consortia have formed to do large-scale analysis of cancer genomes at all different levels of large sets of tumors that will provide essential future information on the landscape of cancer genomes [29].

## Implementation strategies in the clinic

Molecular classification has had limited implementation in standard clinical treatment guidelines [30,31]. There are two molecular signatures that are approved for clinical use in breast cancer; one microarray-based for fresh frozen tumor material (Mammaprint^®^, Agendia, Irvine, CA, USA) and one PCR-based for paraffin embedded tumor material (OncotypeDX^®^, Genomic Health, Inc., Redwood City, CA, USA) [32,33]. The evolving knowledge from molecular classification provides information about disrupted pathways in great detail as well as global changes in expression of genes and genomic alterations. At the same time it is important to acknowledge that existing data for treatment guidelines are based on traditional histopathology and some single molecular markers. To build treatment algorithms that integrate all existing knowledge is currently the challenge.

**Figure 2:**
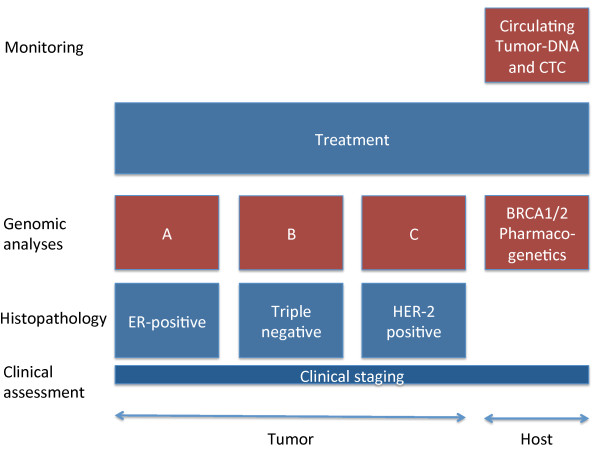
**Outline of implementation strategies in the clinic**. Different layers of assessment of patient and tumor characteristics for treatment decision-making are shown in this schematic figure. The basis is made up of clinical staging and histopathology including important molecular markers, including estrogen (ER) and progesterone receptor (PR), HER-2 and Ki67, to categorize patients into pathological subgroups. As the resulting subgroups are heterogeneous, different molecular assays should be applied within these. Host characteristics important to the choice of treatment and optimization of dosage should be evaluated in parallel. In monitoring treatment response personalized biomarkers should be examined.

We believe that the baseline will still be traditional histopathology combined with clinical staging, but with a second layer of molecular classification with subtype specific prognostic and predictive tests (Figure 2). The heterogeneity of breast cancer makes it likely that different tests should be considered in the different clinical settings. Prognostic tests like MammaPrint or Oncotype DX must be validated for such subgroups of patients and their use must be limited to groups where their prognostic power is validated. Such validation in clinically relevant groups of patients is crucial. Many prognostic signatures are published but inadequate validation makes clinical use futile [34].

At present, patient genotype information is not included in treatment of breast cancer. We indicate in Figure 2 that genotype testing in the future should be included parallel to assessment of the tumor. Germline variation in genes involved in drug metabolism may guide the choice of drugs as well as dosage monitoring, as the influence of CYP2D6 variants on Tamoxifen metabolism [35]. Germline mutations leading to deficient proteins (like BRCA1/2) increase the risk of breast cancer, but can also be exploited in therapy. Cells with deficient BRCA have impaired homologous recombination (HR) and are dependent on alternative DNA repair mechanisms. Inhibition of poly ADP ribose polymerase (PARP) leads to the accumulation of multiple DNA double strand breaks and without efficient repair mechanisms the cell dies [36,37]. Such a synthetic lethality approach is a promising therapeutic strategy.

The highly individualized information provided from deep sequencing has the potential to find individualized biomarkers for treatment and disease monitoring [38,39]. Deep sequencing of single cells will give detailed information about the clonal landscape in tumors [28]. It is likely that clonal diversity affects the response to chemotherapy [40]. Targeted therapy approaches have a great potential in oncology, but resistance to the agents is a clinical problem. In colorectal cancer, it has been shown that treatment with Cetuximab, an inhibitor of EGFR, is ineffective in the presence of an activating mutation of k-ras, a downstream protein in the EGFR signaling pathway [41]. This mechanism of drug resistance is likely to be present for other agents as well.

Deep sequencing of cancer genomes makes it possible to have full mutational information on the important pathways, and methods to characterize the gene sets of mutations are being developed [42,43]. For several of the important carcinogenic pathways several inhibitors exist and more will come. The prospect is, therefore, for better prognostication, prediction and targeted therapy as the main result of full characterization of cancer genomes.

## Conclusion

Results from next generation sequencing have the potential for revolutionizing the understanding of malignant disease. The challenge remains in the integration of new results with existing knowledge based on histopathological stratification of breast cancer.

## Abbreviations

aCGH: array comparative genomic hybridization; CNA: copy number alterations; HR: homologous recombination; PARP: poly ADP ribose polymerase; PCR: polymerase chain reaction.

## Competing interests

CC is a Section Editor for *BMC Cancer*. The authors declare that they have no competing interests.

## Authors' contributions

HKMV and CC wrote the paper. Both authors have read and approved the final manuscript

## Pre-publication history

The pre-publication history for this paper can be accessed here:

http://www.biomedcentral.com/1471-2407/11/501/prepub
